# Leisure Screen Time, Internet Gaming Disorder, and Mental Health Among Chinese Adolescents: Large-Scale Cross-Sectional Study

**DOI:** 10.2196/80737

**Published:** 2026-01-15

**Authors:** Qin Deng, Linna Sha, Jiaojiao Hou, Xunying Zhao, Rong Xiang, Jiangbo Zhu, Yang Qu, Jinyu Zhou, Ting Yu, Xin Song, Sirui Zheng, Tao Han, Bin Yang, Mengyu Fan, Xia Jiang

**Affiliations:** 1 Department of Nutrition and Food Hygiene West China School of Public Health and West China Fourth Hospital Sichuan University Chengdu China; 2 Department of Epidemiology and Biostatistics West China School of Public Health and West China Fourth Hospital Sichuan University Chengdu China; 3 Department of Clinical Neuroscience, Center for Molecular Medicine Karolinska Institutet Stockholm Sweden

**Keywords:** adolescent, cross-sectional study, internet gaming disorder, leisure screen time, mental health, screen media activity

## Abstract

**Background:**

Adolescence is a critical period for mental health vulnerability alongside rising digital media exposure. Current evidence often fails to distinguish the distinct roles of leisure screen time (LST) quantity and addictive patterns like internet gaming disorder (IGD) on a comprehensive range of mental health outcomes.

**Objective:**

This study aimed to investigate the independent and joint associations of LST and IGD with multiple mental health conditions among Chinese adolescents.

**Methods:**

We conducted a school-based, cross-sectional survey in Sichuan Province, China. Participants were recruited by random cluster sampling from 20 public schools. The sample comprised 13,240 adolescents (6659/13,240, 50.3% girls) with a mean age of 15.4 (SD 1.6) years. LST was self-reported, and IGD was evaluated using the Internet Gaming Disorder Scale-9 Item Short Form (IGDS9-SF). Mental health outcomes included overall mental health status and 5 specific diseases: psychological distress, depression, paranoia, insomnia, and suicidal ideation, all assessed using validated scales.

**Results:**

The prevalence of excessive LST, IGD, and any mental health disorder was 48.2% (6378/13,240; 95% CI 47.3%-49.0%), 1.4% (188/13,240; 95% CI 1.2%-1.6%), and 55.8% (7387/13,240; 95% CI 54.9%-56.7%), respectively. After adjustment, excessive LST (odds ratio [OR] 1.18, 95% CI 1.09-1.27) and IGD (OR 6.58, 95% CI 5.02-8.62) were independently associated with poor mental health. A dose-response relationship existed for LST quartiles (Q2: OR 1.15, 95% CI 1.04-1.26; Q3: OR 1.24, 95% CI 1.12-1.37; Q4: OR 1.31, 95% CI 1.18-1.46; *P*_trend_<.001). Excessive LST was associated with depression (OR 1.16, 95% CIs 1.05-1.29), paranoia (OR 1.22, 95% CI 1.11-1.34), and suicidal ideation (OR 1.15, 95% CI 1.04-1.28), while IGD was associated with all 5 disorders, most notably depression (OR 6.43, 95% CI 4.56-9.06) and paranoia (OR 5.77, 95% CI 4.05-8.21). IGD consistently demonstrated stronger associations than LST: psychological distress (OR 4.40, 95% CI 3.12-6.19 vs OR 1.14, 95% CI 0.98-1.33), depression (OR 6.43, 95% CI 4.56-9.06 vs OR 1.16, 95% CI 1.05-1.29), paranoia (OR 5.77, 95% CI 4.05-8.21 vs OR 1.22, 95% CI 1.11-1.34), insomnia (OR 2.90, 95% CI 2.09-4.05 vs OR 1.12, 95% CI 102-1.22), and suicidal ideation (OR 3.85, 95% CI 2.76-5.37 vs OR 1.15, 95% CI 1.04-1.28). Adolescents with both excessive LST and IGD demonstrated the highest odds of mental health disorders (OR 7.35, 95% CI 5.29-10.22). No significant interaction was found on additive or multiplicative scales.

**Conclusions:**

Both excessive LST and IGD are independently associated with mental health disorders in adolescents, with IGD showing a substantially stronger association. This study is distinct from prior research by simultaneously investigating both screen time quantity and addictive usage patterns, and by comprehensively assessing 5 distinct mental health outcomes. Longitudinal studies are needed to better understand the long-term effects.

## Introduction

Adolescence represents a critical period for psychological development, as up to half of all mental health conditions start before age 14 years [1,2]. Globally, 1 in 7 adolescents experience at least one mental health disorder, contributing to 15% of the overall disease burden in this age group [3]. Depression and anxiety are among the leading causes of illness and disability, with suicide being the fourth leading cause of death for those aged 15-19 years [3]. Additionally, psychological distress [4], paranoia [5], and insomnia [6] are also commonly prevalent. Poor mental health at a relatively early stage poses significant adverse effects that can span from the short to the long term [7], including school disengagement, reduced quality of life, and increased mortality. The widespread underrecognition and treatment delays—where only 15.4% of adolescents seek prompt professional help after their initial request—further exacerbate these issues [8].

Alongside the rise in mental health disorders over the years, time spent on screen-based activities continues to increase by as much as 2 hours per day since 2010, particularly among adolescents [9,10]. Data from the Adolescent Brain Cognitive Development Study reveal that adolescents now spend an average of over 5.5 hours daily on noneducational screen media, underscoring a global trend of pervasive digital engagement [11]. While moderate screen use may offer cognitive benefits such as enhanced multitasking [12], excessive use is linked to sedentary behaviors, impaired parent–child interactions, and behavioral addictions [13,14]. Each additional hour of daily screen time in late childhood predicted increased depressive symptoms in early adolescence, an effect mediated by shorter sleep duration and changes in white matter organization in brain regions responsible for emotion regulation [15]. Current research often conflates educational and leisure screen time (LST), obscuring the unique risk of LST [16,17]. A dose-response study of Chinese adolescents revealed that LST exceeding 1 hour/day linearly increases mental health problems, with video-based and gaming content showing the strongest negative associations [18]. Nevertheless, existing studies have mainly investigated depression and anxiety, the 2 most prevalent mental health issues, while largely disregarding other important disorders such as paranoia and insomnia that affect a nontrivial proportion (20%-30%) of adolescents [5,17].

The amount of leisure time spent using screen-based media does not necessarily represent the extent of harm. Crucially, recent research underscores that addictive use patterns—characterized by compulsive use, loss of control, and continued use despite negative consequences—may represent a far greater risk than screen time duration alone [14]. Studies found that a small number of individuals, despite showing shorter screen time, exhibited characteristics of addictive behaviors [19]. A primary concern for adolescents is gaming addiction, particularly the internet gaming disorder (IGD), defined by *DSM-5* (*Diagnostic and Statistical Manual of Mental Disorders* [Fifth Edition]) as the persistent and recurrent use of internet to play games, often with others. IGD leads to clinically significant impairment or distress and has been listed as a tentative disorder in need of further study [20]. IGD has been linked to distinct neurobiological changes and impairments in executive function, underscoring its status as a behavioral addiction beyond mere excessive use [21]. Interestingly, a substantial proportion of individuals with long screen time do not meet the symptomatic criteria of IGD, while those who meet criteria do not necessarily show long screen time [22,23]. This dissociation underscores the need to examine both behaviors simultaneously.

The interplay between LST and IGD in relation to a broad spectrum of mental health outcomes remains inadequately explored. While longitudinal evidence suggests that internalizing symptoms (eg, depression, anxiety) fully mediate the link between problematic internet use and subsequent self-harm behaviors, this model did not account for the joint influence of general screen time [24]. Furthermore, seminal research has demonstrated that trajectories of addictive digital media use are independently associated with heightened risks of suicidality, regardless of baseline screen time [14]. Moreover, while high LST and IGD independently poses negative effects on mental health, it is likely that those who exhibit both behaviors are at the highest risk. However, few studies have analyzed leisure and addictive screen-based media use by taking into consideration their independent and joint effects on mental health outcomes.

This cross-sectional study aims to comprehensively investigate the independent and joint associations of LST and IGD with an array of mental health among Chinese adolescents, including overall mental health and its 5 major diseases, namely, depression, psychological distress, paranoia, insomnia, and suicidal ideation.

## Methods

### Ethical Considerations

The research was conducted in accordance with the Declaration of Helsinki and received approval from the Ethics Committee of West China Fourth Hospital and West China School of Public Health, Sichuan University (Gw112023133; June 13, 2023). Written informed consent was obtained from parents or guardians after they received a detailed information sheet outlining the study purpose, procedures, and voluntary nature of participation. Student assent was also secured at the time of the survey. No compensation was offered to participants for their involvement in this study. Beyond a lack of financial compensation, the study also prioritized participant anonymity by eliminating all personally identifiable information from the collected data.

Additionally, this study did not collect any personally identifiable information, such as facial images, voice recordings, or other biometric data. All data presented in this manuscript and supplementary materials are fully anonymized, ensuring that no individual participant can be identified.

### Study Population

This cross-sectional study analyzed data from a survey project on the mental health of children and adolescents in Pidu District, Chengdu, conducted from June to December 2023 by the West China School of Public Health and the West China Fourth Hospital, Sichuan University. We adhered to the STROBE (Strengthening the Reporting of Observational Studies in Epidemiology) guidelines in reporting cross-sectional studies [25].

The survey aimed to gain a comprehensive understanding of the mental health status of children and adolescents residing in Pidu District and to effectively address the mental health issues affecting this population. To ensure the representativeness of study samples, we used a random cluster sampling design based on the number and proportion of primary and secondary students in Pidu District, considering types of school and their urban–rural distribution. A total of 20 schools were selected, comprising 17,973 registered students for the questionnaire survey.

Data collection was conducted in classroom settings during regular school hours. Trained research staff administered structured, paper-based questionnaires to all participants. To ensure standardized procedures, staff delivered standardized verbal instructions to students before distributing the questionnaires, detailing the study’s purpose, its voluntary nature, confidentiality protocols, and questionnaire completion guidelines. Participants then completed the surveys independently. Research staff supervised the process and were available to provide neutral clarification on questions but refrained from influencing participant responses. Of the 16,482 participants invited, a total of 16,325 were present on the survey day and completed the questionnaire, resulting in a response rate of 98.99%. Participant eligibility was restricted to Han Chinese adolescents aged 12-18 years. Ethnicity was self-identified in the questionnaire, and age was calculated from the self-reported date of birth and ensured to be within the specified range at the time of data collection. After applying the eligibility criteria, a total of 13,240 participants were included in the final analytical sample. The high response rate minimized concerns about selection bias.

### Assessment of LST and IGD

LST was measured using a self-reported questionnaire. Participants were asked to recall and report the average time they spent on leisure screen activities (including entertainment-based use of smartphones, computers, tablets, and televisions) on a typical day. The average daily LST was calculated using the formula: ([Weekday LST × 5] + [Weekend LST × 2])/7. In this study, LST was categorized into 2 groups according to World Health Organization standards, low (<2 h/day) and high (≥2 h/day) [26], and further into 4 groups based on quartiles, Q1 (≤0.93 h/day), Q2 (0.93 <LST ≤1.86 h/day), Q3 (1.86 <LST ≤4.19 h/day), and Q4 (>4.19 h/day).

IGD was assessed using the Internet Gaming Disorder Scale-9 Item Short Form (IGDS9-SF) [27], consisting of 9 items reflecting the diagnostic criteria for IGD as defined by the American Psychological Association. Participants responded to each item through a Likert scale ranging from 1 (“never”) to 5 (“very often”). Total scores, ranging from 9 to 45, were calculated by summing across the responses. This threshold was identified as clinically optimal for the Chinese population in a previous validation study [28], which, although conducted in a sample with a mean age of 20 years, remains one of the most established cutoffs for the IGDS9-SF in the absence of a universally validated threshold for early- to mid-adolescents. We selected this cutoff to ensure consistency with prior research and because of its high specificity, which minimizes false positives. Furthermore, the scale demonstrated adequate internal consistency in our adolescent sample (Cronbach α=0.88), supporting its reliability for this age group.

To identify the joint association of the 2 types of screen-based behaviors, we defined a combined pattern based on the level of LST (low vs high) and status of IGD (absence vs presence). We designated the group with LST of less than 2 hours and without IGD as the reference group, referred to as “Low No.” The other 3 comparison groups were “High No,” “Low Yes,” and “High Yes.”

### Assessment of Mental Health Disorders

We applied well-established validated scales to investigate the 5 common mental health disorders among adolescents. Specifically, psychological distress was measured using the Kessler 6-item (K6) scale, which assesses the frequency of psychopathological symptoms or behaviors. A K6 score of ≥13 indicates psychological distress [29,30]. Depression was evaluated using the Kutcher Adolescent Depression 6-item (KADS-6) scale, specifically designed for children and adolescents to effectively identify major depressive episodes. A KADS-6 score of ≥6 indicates depression [31]. Paranoia was assessed using the psychoticism and paranoid ideation subscales of the Symptom Checklist-90 (SCL-90), with a score of ≥12 indicating paranoid ideation [32]. Insomnia was measured using the Insomnia Severity Index (ISI) scale, with a score of ≥8 indicating sleep disturbances [33]. Suicidal ideation was assessed using the Suicide Behaviors Questionnaire-Revised (SBQ-R) scale, with a score of ≥8 reflecting risk of suicidal behaviors [34]. Adolescents exhibiting any (or more) of these 5 symptoms were considered to have mental health disorders. The internal consistency of all scales used in this study appeared to be robust, with Cronbach α values being 0.88 (K6), 0.85 (KADS-6), 0.81 (SCL-90), 0.88 (ISI), and 0.84 (SBQ-R), respectively.

### Assessment of Covariates

This analysis included a comprehensive set of variables that may potentially confound the association between screen-based behaviors and adolescent mental health (Table S1 in Multimedia Appendix 1). Demographic variables included age, sex, area of residence, economic status, single-child status, type of caretakers, and parental educational level. The area of residence was classified as “urban” or “rural.” Economic status was categorized into “high-income” or “low- to middle-income.” Single-child status was determined based on the presence of siblings (“yes” or “no”). Caretaker type was classified as “both parents and others” or “one parent.” Parental educational level was defined by the highest education attained: “junior school and below,” “high school or vocational,” and “college and above.” Anthropometric measures included BMI (kg/m²), calculated from height and weight. Lifestyle factors included smoking status, drinking status, academic performance, physical health status, dietary habits, and physical activity (PA) level. Smoking and drinking were self-reported as “yes” or “no.” Academic performance and physical health status were categorized as “low,” “moderate,” or “high.” Dietary habits—including breakfast, vegetables and fruits, protein consumption, sugary beverages, desserts, and fried food—were divided into “never eat,” “1-2 times per week,” “3-4 times per week,” “5-6 times per week,” and “eat every day.” PA levels were categorized based on daily durations into 4 quartiles: Q1 (≤1.80 hours), Q2 (1.81-4.80 hours), Q3 (4.81-9.50 hours), and Q4 (>9.50 hours).

### Statistical Analysis

The missing data pattern was evaluated using the Little’s Missing Completely at Random test. A nonsignificant result (*χ*²_8836_=4638.4; *P*>.05) confirmed that the data were missing completely at random, thereby justifying the application of multiple imputation. Consequently, the Multiple Imputation by Chained Equations approach was used to handle the missing values, given the low overall missing rate (maximum: 5.7%) [35]. We generated m=5 imputed datasets using predictive mean matching with a maximum of maxit=50 iterations, considered sufficient to achieve stability for datasets with low missing rates. Convergence was assessed by ensuring the algorithm completed the specified iterations without issues. To validate the imputation, we conducted analyses using both raw and imputed datasets, considering results statistically significant only if they were consistent across both methods. Descriptive summaries were presented as mean (SD) for continuous variables and as quantity (proportion) for categorical variables. Differences in continuous and categorical variables between groups (individuals with or without mental health disorders) were assessed using *t* tests (2-tailed) and chi-square tests, as appropriate.

To assess multicollinearity between LST and IGD, we used Pearson correlation coefficients (*r*) with values greater than 0.5 and variance inflation factor values exceeding 10 as diagnostic tests. The variance inflation factor value was found to be less than 1.02, indicating that multicollinearity was of minimal concern.

Multivariable logistic regression was used to examine the associations between screen-based behaviors and mental health among adolescents, controlling for confounders. First, we assessed the independent association of LST (high vs low) and IGD (presence vs absence) with overall mental health condition, making mutual adjustment for each other. Meanwhile, we also categorized LST into quartiles based on its duration (Q1-Q4) and used the Cochran-Armitage test to evaluate its dose-response effect [36]. Second, we investigated the joint effects and interactions of LST and IGD on overall mental health condition, adopting the Cochran-Armitage test to evaluate a linear trend. Finally, subgroup analyses were conducted by sex, area, economic status, single-child status, and type of caretakers. Interaction between screen-based behaviors and stratification factors was performed using the likelihood ratio test comparing models with and without a cross-product term [37]. Such a comprehensive analytical framework was further applied in parallel to each specific mental health disorder. In addition, to account for the cluster sampling design (participants nested within 20 schools), we used multilevel mixed-effects models with random intercepts for schools as sensitivity analyses.

Statistical analysis were performed using R (version 4.3.3; The R Core Team). Association estimates were presented in the form of odds ratios (ORs) and their 95% CIs. To minimize type I error, Bonferroni correction was applied, with a 2-sided *P* value <.05/5 considered statistically significant for the 5 mental health disorders. Trend tests and interaction *P* values were defined as statistically significant at *P*<.05.

## Results

### Participant Characteristics

The main characteristics of participants according to the status of mental health disorders are presented in Table S1 in Multimedia Appendix 1. This study included 13,240 adolescents (6659/13,240, 50.3% girls) with a mean age of 15.4 (SD 1.6) years. Among these adolescents, 60% (7947/13,240; 95% CI 59.1%-60.9%) lived in the rural areas, and a majority (12,264/13,240, 92.5%; 95% CI 92.0%-93.0%) were from low- to middle-income families. More than half of the participants (8543/13,240, 64%; 95% CI 63.2%-64.8%) had no siblings, and a significant proportion of parents (55.3%-58.9%) received low levels of education.

Regarding the outcomes, a total of 7391 adolescents (55.8%; 95% CI 54.9%-56.7%) were identified as having at least 1 of the 5 mental health disorders. Insomnia was the most prevalent, affecting 35.3% (4677/13,240; 95% CI 34.5%-36.1%) of participants, followed by paranoia (3670/13,240, 28.4%; 95% CI 27.6%-29.2%), suicidal ideation (3155/13,240, 23.8%; 95% CI 23.0%-24.6%), depression (2733/13,240, 20.6%; 95% CI 19.9%-21.3%), and psychological distress (1091/13,240, 8.2%; 95% CI 7.7%-8.7%).

Regarding the exposures, a total of 6378 adolescents (48.2%; 95% CI 47.3%-49.0%) had LST exceeding 2 hours per day, and 188 individuals (1.4%; 95% CI 1.2%-1.6%) met the criteria for IGD. Notably, among those with IGD (n=188), a total of 58 individuals (30.9%; 95% CI 24.3%-38.1%) had LST of 2 hours or less.

The prevalence of any mental health condition was higher among females, those with single parents or other caretakers, those with low academic performance, smokers, drinkers, those with poor physical health, those who rarely consumed breakfast, fruits, and vegetables or protein, as well as those who frequently consumed unhealthy foods (sugary beverages, sweets, and fried food) (*P*<.05 for all).

### Independent Association of LST and IGD on Mental Health Conditions

Results on the associations of each specific screen-based behavior with mental health disorders are shown in Table 1. After adjusting for potential confounders, LST >2 hours/day (OR 1.18, 95% CI 1.09-1.27) and the presence of IGD (OR 6.58, 95% CI 5.02-8.62) were significantly and independently associated with poorer mental health status. In addition, the odds of overall mental health issues showed a marked increase as LST increased (Q2: OR 1.15, 95% CI 1.04-1.26; Q3: OR 1.24, 95% CI 1.12-1.37; Q4: OR 1.31, 95% CI 1.18-1.46; *P*_trend_<.001) (Table S2 in Multimedia Appendix 1).

**Table 1:** Independent associations of leisure screen time and internet gaming disorder with mental health disorders among Chinese adolescents (*P*<.05/5).

Mental health outcome		Leisure screen time (h/day)					Internet gaming disorder			
		<2	≥2			No			Yes	
		OR^a,b^	OR (95% CI)	*P* value	OR^c^			OR (95% CI)		*P* value
**Overall mental health**										
	Model 1^d^	Ref	1.56 (1.45-1.67)	<.001	Ref			11.24 (8.69-14.55)		<.001
	Model 2^e^	Ref	1.19 (1.11-1.28)	<.001	Ref			6.68 (5.10-8.75)		<.001
	Model 3^f^	Ref	1.18 (1.09-1.27)	<.001	Ref			6.58 (5.02-8.62)		<.001
**Psychological distress**										
	Model 1	Ref	1.61 (1.40-1.85)	<.001	Ref			7.86 (5.77-10.71)		<.001
	Model 2	Ref	1.17 (1.01-1.37)	.04	Ref			4.47 (3.18-6.29)		<.001
	Model 3	Ref	1.14 (0.98-1.33)	.09	Ref			4.40 (3.12-6.19)		<.001
**Depression**										
	Model 1	Ref	1.57 (1.43-1.73)	<.001	Ref			9.98 (7.29-13.66)		<.001
	Model 2	Ref	1.19 (1.07-1.32)	.002	Ref			6.52 (4.63-9.18)		<.001
	Model 3	Ref	1.16 (1.05-1.29)	.005	Ref			6.43 (4.56-9.06)		<.001
**Paranoia**										
	Model 1	Ref	1.52 (1.39-1.65)	<.001	Ref			8.38 (5.99-11.72)		<.001
	Model 2	Ref	1.24 (1.13-1.36)	<.001	Ref			5.88 (4.13-8.36)		<.001
	Model 3	Ref	1.22 (1.11-1.34)	<.001	Ref			5.77 (4.05-8.21)		<.001
**Insomnia**										
	Model 1	Ref	1.41 (1.30-1.53)	<.001	Ref			4.34 (3.18-5.93)		<.001
	Model 2	Ref	1.13 (1.03-1.23)	.01	Ref			2.94 (2.11-4.09)		<.001
	Model 3	Ref	1.12 (1.02-1.22)	.01	Ref			2.90 (2.09-4.05)		<.001
**Suicidal ideation**										
	Model 1	Ref	1.52 (1.39-1.67)	<.001	Ref			6.33 (4.67-8.58)		<.001
	Model 2	Ref	1.17 (1.06-1.29)	.003	Ref			3.90 (2.80-5.45)		<.001
	Model 3	Ref	1.15 (1.04-1.28)	.01	Ref			3.85 (2.76-5.37)		<.001

^a^OR: odds ratio.

^b^The reference group for leisure screen time was <2 h/day.

^c^The reference group for internet gaming disorder was “No.”

^d^Model 1 (partially adjusted model) was adjusted for age, sex, area, and economic status.

^e^Model 2 (fully adjusted model) was adjusted for age, sex, area, economic status, single child, caretaker, father’s educational level, mother’s educational level, BMI, smoking, drinking, academic performance, health status, dietary factors, and physical activity level.

^f^Model 3 adjusted for the covariates included in Model 2 and additionally included mutual adjustment for leisure screen time and internet gaming disorder.

For specific disorders, excessive LST was associated with significantly higher odds of depression (OR 1.16, 95% CI 1.05-1.29), paranoia (OR 1.22, 95% CI 1.11-1.34), and suicidal ideation (OR 1.15, 95% CI 1.04-1.28). Similarly, IGD was significantly associated with psychological distress (OR 4.40, 95% CI 3.12-6.19), depression (OR 6.43, 95% CI 4.56-9.06), paranoia (OR 5.77, 95% CI 4.05-8.21), insomnia (OR 2.90, 95% CI 2.09-4.05), and suicidal ideation (OR 3.85, 95% CI 2.76-5.37).

**Figure 1:**
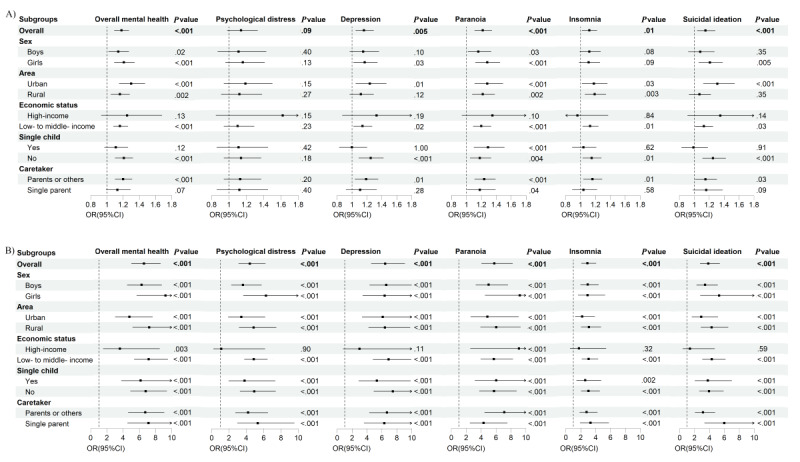
Subgroup analysis of the associations of screen-based behaviors with mental health disorders in Chinese adolescents. (A) Association between leisure screen time (LST; h/day) and mental health; (B) association between internet gaming disorder (IGD) and mental health.

In subgroup analysis, no factor was found to significantly modify the associations between LST and any mental health condition, nor between IGD and any mental health condition (all *P*_interaction_>.05) (Figure 1 and Tables S7 and S8 in Multimedia Appendix 1).

### Joint Associations of LST and IGD on Mental Health Conditions

**Figure 2:**
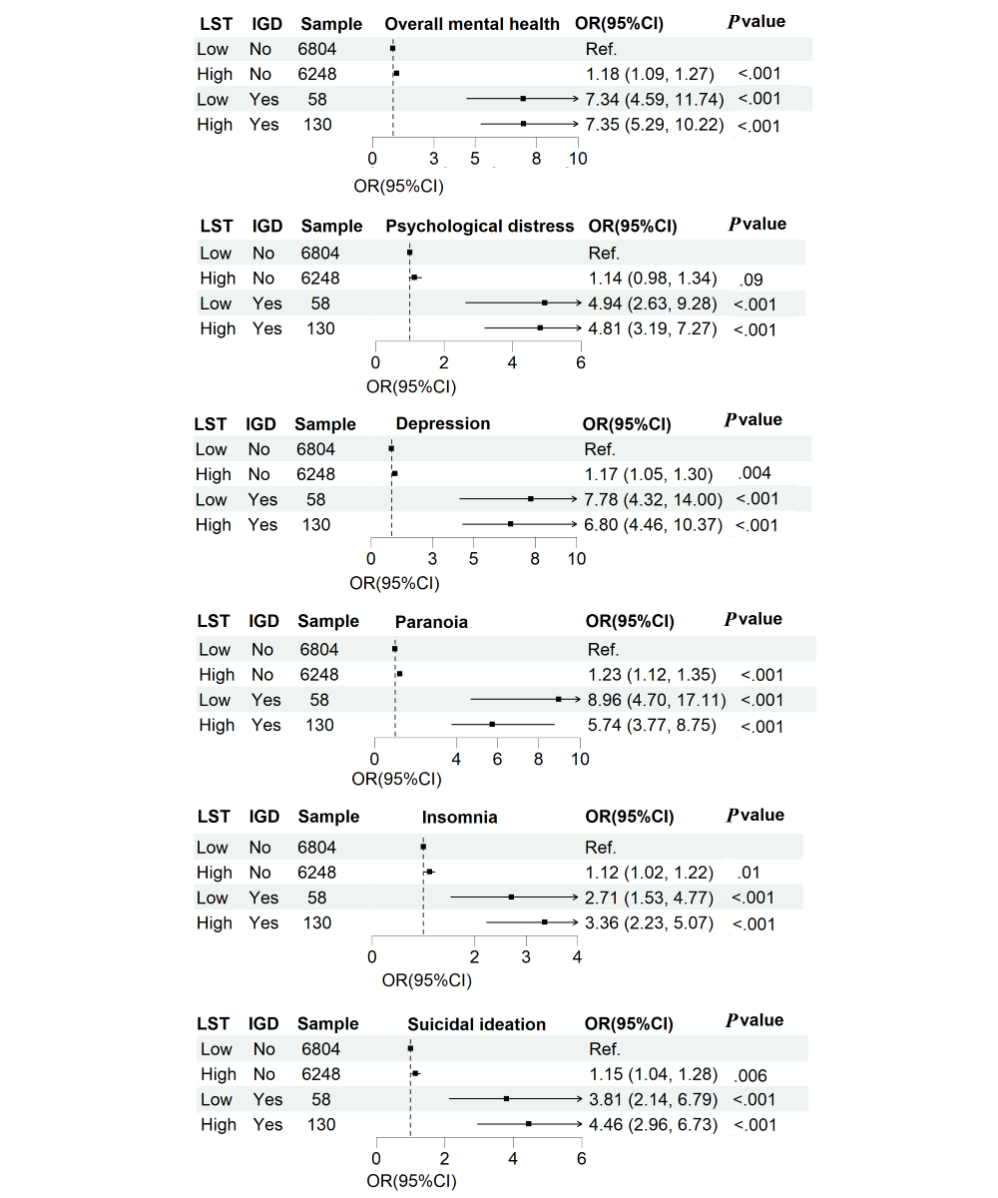
Combined effect of leisure screen time (LST) and internet gaming disorder (IGD) on mental health disorders among Chinese adolescents.

We further examined the joint associations of LST and IGD with the outcomes, as presented in Table 2 and Figure 2. The odds of overall mental health conditions were significantly higher among adolescents who exhibited at least one type of screen-based behavior (High No: OR 1.18, 95% CI 1.09-1.27; Low Yes: OR 7.34, 95% CI 4.59-11.74). Notably, the highest odds were observed when both excessive LST and symptoms of IGD were present simultaneously (High Yes: OR 7.35, 95% CI 5.29-10.22). However, no significant interaction between LST and IGD, either on the additive scale (relative excess risk due to interaction=–0.77, 95% CI –22.05 to 17.02; attributable proportion=–0.01, 95% CI –2.86 to 0.69; synergy index=0.99, 95% CI 0.23-4.26) or on the multiplicative scale (OR 0.88, 95% CI 0.23-3.37), was observed (Table 3). A similar pattern of results was observed with each of the 5 individual mental health outcomes.

**Table 2:** Combined effect of leisure screen time (LST) and internet gaming disorder (IGD) on mental health (*P*<.05/5). Odds ratios (ORs) of the joint associations were not directly comparable to those of the independent associations as shown in as they were derived from different models with different reference groups and adjustment strategies.

Mental health outcome	LST_IGD							
	Low No	High No		Low Yes			High Yes	
	OR^a^	OR (95% CI)	*P* value	OR (95% CI)	*P* value	OR (95% CI)		*P* value
Overall mental health	Ref	1.18 (1.09-1.27)	<.001	7.34 (4.59-11.74)	<.001	7.35 (5.29-10.22)		<.001
Psychological distress	Ref	1.14 (0.98-1.34)	.09	4.94 (2.63-9.28)	<.001	4.81 (3.19-7.27)		<.001
Depression	Ref	1.17 (1.05-1.30)	.004	7.78 (4.32-14.00)	<.001	6.80 (4.46-10.37)		<.001
Paranoia	Ref	1.23 (1.12-1.35)	<.001	8.96 (4.70-17.11)	<.001	5.74 (3.77-8.75)		<.001
Insomnia	Ref	1.12 (1.02-1.22)	.01	2.71 (1.53-4.77)	<.001	3.36 (2.23-5.07)		<.001
Suicidal ideation	Ref	1.15 (1.04-1.28)	.006	3.81 (2.14-6.79)	<.001	4.46 (2.96-6.73)		<.001

^a^Adjusted for age, sex, area, economic status, single child, caretaker, father’s educational level, mother’s educational level, BMI, smoking, drinking, academic performance, health status, dietary factor and physical activity level.

**Table 3:** Interaction analysis between leisure screen time and internet gaming disorder on mental health disorders among Chinese adolescents.

Mental health outcome	Additive interaction				Multiplicative interaction
	RERI^a^ (95% CI)	AP^b^ (95% CI)	S^c^ (95% CI)	OR^d,e^ (95% CI)	
Overall mental health	–0.77 (–22.05 to 17.02)	–0.01 (–2.86 to 0.69)	0.99 (0.23 to 4.26)	0.88 (0.23 to 3.37)	
Psychological distress	–0.27 (–4.83 to 3.00)	–0.06 (–1.20 to 0.47)	0.93 (0.38 to 2.30)	0.85 (0.40 to 1.79)	
Depression	–1.14 (–7.73 to 3.75)	–0.17 (–1.38 to 0.41)	0.84 (0.37 to 1.88)	0.75 (0.36 to 1.54)	
Paranoia	–3.45 (–11.80 to 1.73)	–0.60 (–2.43 to 0.22)	0.58 (0.24 to 1.37)	0.52 (0.24 to 1.12)	
Insomnia	0.54 (–1.80 to 2.58)	0.16 (–0.69 to 0.54)	1.30 (0.47 to 3.58)	1.11 (0.55 to 2.23)	
Suicidal ideation	0.50 (–2.81 to 3.28)	0.11 (–0.79 to 0.53)	1.17 (0.47 to 2.88)	1.17 (0.47 to 2.88)	

^a^RERI: relative excess risk due to interaction.

^b^AP: attributable proportion due to interaction.

^c^S: synergy index.

^d^OR: odds ratio.

^e^Adjusted for age, sex, area, economic status, single child, caretaker, father’s educational level, mother’s educational level, BMI, smoking, drinking, academic performance, health status, dietary factor and physical activity level.

Repeating all analyses using raw data, we obtained consistent findings (Tables S4-S6 and S9-S12 in Multimedia Appendix 1). Sensitivity analyses using multilevel models accounting for school clustering yielded results consistent with primary findings, with all significant associations remaining unchanged. Intraclass correlation coefficients (0.76%-2.08%) confirmed that most variance (>98%) occurred at the individual level, supporting the robustness of results (Tables S18 and S19 in Multimedia Appendix 1).

## Discussion

### Principal Findings

In this large-scale cross-sectional study of more than 10,000 Chinese adolescents, 48.2% (6378/13,240) of the study population reported excessive LST, and 1.4% (188/13,240) met the criteria for IGD. Both excessive LST (>2 h/day) and IGD were independently associated with increased odds of overall mental health disorders and its 5 major conditions: psychological distress, depression, paranoia, insomnia, and suicidal ideation. The association was markedly stronger for IGD than for LST. The observed dose-response relationship across LST quartiles, with progressively higher ORs across LST quartiles, provides compelling evidence for a graded association in which increasing screen time exposure corresponds to incrementally worse mental health outcomes. Furthermore, while a significant joint association was observed—where adolescents exhibiting both behaviors demonstrated a 7-fold rise in the probability of mental health disorders—no statistically significant interaction was found, indicating an additive rather than synergistic effect. The precision of the main effect estimates further strengthens the robustness of our findings.

These findings necessitate an expansion of current public health guidelines, which predominantly target screen time duration, to include specific strategies for early identification and management of IGD. This study makes a critical contribution by clearly establishing IGD as a severe and distinct factor, independent of mere usage quantity. Methodologically, the innovative joint-association analysis revealing their additive relationship provides a more sophisticated framework for assessing digital media behaviors on mental health.

### Prevalence of LST and IGD Among Adolescents

Approximately 48.2% (6378/13,240) of adolescents in this study reported LST >2 hours/day, surpassing the rate reported in a recent study conducted in Guangzhou, China (20.7%) [38]. The prevalence of IGD in this study was only 1.4% (188/13,240), much lower than the global rate of 8.8% [39]. Such discrepancy may be attributed to several factors. First, it likely reflects our use of a conservative, high-specificity cutoff score (≥32 on IGDS9-SF) to minimize false positives, whereas many epidemiological studies use lower, more sensitive thresholds that yield higher prevalence rates. Second, our sample was drawn from a general school-based population in a specific sociocultural context (Pidu District, Chengdu), which may exhibit different characteristics compared to the more diverse and potentially higher-risk samples included in the global meta-analyses.

Despite the lower prevalence, the large overall sample size (N=13,240) ensured that this study was sufficiently powered to detect strong and statistically significant associations, as evidenced by the strength and consistency of the association between IGD and poorer mental health. It is also important to note that the large population size of China means that even a 1.4% prevalence translates to a substantial absolute number of adolescents affected by IGD, underscoring the importance for policymakers and guardians to closely monitor screen-based behaviors among adolescents. Of note, we found that a large proportion of adolescents meeting the symptomatic criteria for IGD (58/188, 30.9%) reported LST of less than 2 hours/day; conversely, a substantial proportion of participants who did not meet the criteria for IGD (6248/13,052, 47.9%) reported excessive LST. These findings further illustrate that spending a large amount of time on gaming or using media does not necessarily equal to addictive use, and vice versa.

Adolescents living with a single parent or without siblings were more likely to engage in excessive LST. This may stem from reduced supervision in single-parent households or absence of siblings that limit the opportunities for outdoor or other non–screen-based activities, thereby contributing to excessive LST. In addition, boys were more likely than girls to exhibit symptoms of IGD. Boys typically prefer to participate in active and combative activities, whereas girls tend to engage more in social and conversational activities. These preferences may explain why boys are more often drawn to the virtual worlds of online games [40].

### The Association of LST and IGD With Mental Health Conditions

Our findings indicated that excessive LST and IGD, both independently and jointly, were associated with mental health disorders. Prior studies showed that greater time spent on screen media was correlated with a higher prevalence of multiple mental health disorders, such as depression and psychological distress [41-43]. This study extends the existing body of work by providing new evidence for additional mental health outcomes, including paranoia and suicidal ideation in adolescents.

One possible explanation is that screen-based activities displace time for other beneficial activities, such as PA [44], a well-established factor protecting against mental health disorders [45,46]. Indeed, previous studies have demonstrated that PA can help reduce excessive screen-based behaviors and improve mental health. Since PA and screen-based behaviors often co-develop during adolescence [47], fostering healthy lifestyles is crucial for both the current and the future well-being of this population. Another key factor is the inherent harm posed by screen-based behaviors. Beyond educational content, the internet and media are flooded with violent, graphic, and pornographic materials, which further exacerbate mental health [48].

Adolescents with both high levels of LST and symptoms of IGD had significantly higher odds of mental health issues compared to those with excessive LST or IGD alone. These behaviors may reinforce each other: high LST could increase the likelihood of developing IGD, while IGD, in turn, may encourage further screen time, creating a feedback loop that exacerbates mental health. Despite such co-occurrence, the absence of significant interactions, neither additive nor multiplicative, suggests that the associations of LST and IGD with mental health are independent rather than synergistic.

This conclusion is supported by the nonsignificant interaction, indicating a lack of synergy where the joint presence does not create a disproportionately greater association. In other words, while the combination of high LST and IGD was associated with a higher likelihood of mental health problems, their interaction did not statistically amplify or mitigate the effect of each behavior. Notably, the group with IGD but low LST (“Low Yes”) exhibited substantially higher odds of mental health disorders than the group with high LST but no IGD (“High No”). This indicates that while both behaviors warrant attention, IGD constitutes a disproportionately strong associated factor. Therefore, future research and public health interventions should address both factors, prioritizing the identification and management of IGD within a framework that includes multidimensional assessments of both screen time and gaming disorder symptoms.

Although no significant modifier was found to influence the associations between LST and mental health disorders, our findings suggested that the association was somewhat more pronounced in girls. Consistent with previous studies, girls tend to spend more time on screen-based media than boys [49,50] and report poorer mental health outcomes [51]. Subgroup analyses revealed distinct patterns of vulnerability: urban residents, adolescents from higher-income families, nonsingle children, and those living with parents or caregivers showed stronger associations between excessive LST and mental health problems. In contrast, the link between IGD and mental health issues was more pronounced among girls, nonsingle children, rural adolescents, individuals from low- to middle-income families, and those in single-parent households. According to a report released by the China Internet Network Information Center, while internet penetration rates among Chinese adolescents are comparable across urban and rural areas, differences emerge in the use of specific online applications [52,53]. Moreover, previous research has shown that parenting style and family function play a pivotal role in the development of screen-based media dependency [54].

Evidence also indicates that parental absence can contribute to depression in children [55], potentially due to disruptions in child–parent relationships resulting from parental migration [56] and reduced communication [57]. These factors may influence the impact of LST and IGD on mental health, as variations in family dynamics and parenting styles affect how screen-based behaviors influence emotional and psychological outcomes. For instance, supportive family environments may mitigate the negative effects of excessive screen time while dysfunctional family interactions could exacerbate the associated negative outcomes.

### Limitations

Regarding the limitations, first, due to the cross-sectional design of this study, it is not possible to establish causal relationships between exposure and outcomes. Second, although random cluster sampling was used, our participants were exclusively Han Chinese adolescents from Pidu District, Chengdu. While this sample captured urban–rural diversity within the district, it may not fully represent the broader socioeconomic, cultural, and regional heterogeneity across China. Therefore, caution is warranted when extrapolating our findings to inform national-level policies, and they should be interpreted as preliminary evidence primarily relevant to similar regional contexts. Third, a key limitation is that all data, including parameters for LST, IGD, and mental health symptoms, were based on self-report. This may introduce reporting bias. To address these limitations, future research could consider using longitudinal and interventional studies to validate the detrimental effects of screen-based behaviors on adolescent mental health. Expanding the research scope to include adolescents from multiple regions might enhance the representativeness and generalizability of findings. Additionally, using objective measurement tools, such as wearable devices or monitoring instruments, for screen time, alongside clinical interviews for diagnosing IGD and mental health disorders, would greatly improve the objectivity and accuracy of the assessments.

### Conclusions

This large-scale population-based study provides novel evidence that LST and IGD represent distinct dimensions of digital engagement with independent and additive associations with mental health problems in Chinese adolescents. Methodologically, this study advances the field by simultaneously examining both screen time quantity and addictive usage patterns, using a comprehensive assessment of 5 mental health outcomes often overlooked in previous research. These findings highlight the need to expand public health strategies beyond screen time limits to include early IGD screening, emphasizing that both the quantity and quality of digital engagement require consideration. Future longitudinal studies should confirm these relationships and inform targeted interventions.

## Appendix

Multimedia Appendix 1 Supplementary tables.

## Abbreviations

DSM-5: Diagnostic and Statistical Manual of Mental Disorders (Fifth Edition)
IGD: internet gaming disorder
IGDS9-SF: Internet Gaming Disorder Scale-9 Item Short Form
ISI: Insomnia Severity Index
K6: Kessler 6-item
KADS-6: Kutcher Adolescent Depression 6-item
LST: leisure screen time
OR: odds ratio
PA: physical activity
SBQ-R: Suicide Behaviors Questionnaire-Revised
SCL-90: Symptom Checklist-90
STROBE: Strengthening the Reporting of Observational Studies in Epidemiology

## Data Availability

The datasets generated and/or analyzed during this study are not publicly available, but can be obtained from the corresponding author upon reasonable request.
